# Photochemical Internalization of Etoposide Using Dendrimer Nanospheres Loaded with Etoposide and Protoporphyrin IX on a Glioblastoma Cell Line

**DOI:** 10.3390/pharmaceutics13111877

**Published:** 2021-11-05

**Authors:** Martin Hsiu-Chu Lin, Li-Ching Chang, Chiu-Yen Chung, Wei-Chao Huang, Ming-Hsueh Lee, Kuo-Tai Chen, Ping-Shan Lai, Jen-Tsung Yang

**Affiliations:** 1 Department of Neurosurgery, Chang Gung Memorial Hospital, Chia-Yi Branch, Chia-Yi 61363, Taiwan; m7099@cgmh.org.tw (M.H.-C.L.); yen16@cgmh.org.tw (C.-Y.C.); u9201031@cgmh.org.tw (W.-C.H.); ming2072@cgmh.org.tw (M.-H.L.); tad91116@cgmh.org.tw (K.-T.C.); 2 Department of Chemistry, National Chung Hsing University, Taichung 402, Taiwan; pslai@email.nchu.edu.tw; 3 Ph.D. Program in Tissue Engineering and Regenerative Medicine, Biotechnology Center, National Chung Hsing University, Taichung 402, Taiwan; 4 Department of Dentistry, Chang Gung Memorial Hospital, Chia-Yi Branch, Chia-Yi 61363, Taiwan; liching@ms39.hinet.net; 5 Department of Nursing, Chang Gung University of Science and Technology, Chia-Yi 61363, Taiwan; 6 College of Medicine, Chang Gung University, Tao-Yuan 33302, Taiwan

**Keywords:** etoposide, protoporphyrin IX, phototherapy, dendrimer, glioblastoma multiforme

## Abstract

Glioblastoma multiforme (GBM) is the most common malignant primary neoplasm of the adult central nervous system originating from glial cells. The prognosis of those affected by GBM has remained poor despite advances in surgery, chemotherapy, and radiotherapy. Photochemical internalization (PCI) is a release mechanism of endocytosed therapeutics into the cytoplasm, which relies on the membrane disruptive effect of light-activated photosensitizers. In this study, phototherapy by PCI was performed on a human GBM cell-line using the topoisomerase II inhibitor etoposide (Etop) and the photosensitizer protoporphyrin IX (PpIX) loaded in nanospheres (Ns) made from generation-5 polyamidoamine dendrimers (PAMAM(G5)). The resultant formulation, Etop/PpIX-PAMAM(G5) Ns, measured 217.4 ± 2.9 nm in diameter and 40.5 ± 1.3 mV in charge. Confocal microscopy demonstrated PpIX fluorescence within the endo-lysosomal compartment, and an almost twofold increase in cellular uptake compared to free PpIX by flow cytometry. Phototherapy with 3 min and 5 min light illumination resulted in a greater extent of synergism than with co-administered Etop and PpIX; notably, antagonism was observed without light illumination. Mechanistically, significant increases in oxidative stress and apoptosis were observed with Etop/PpIX-PAMAM(G5) Ns upon 5 min of light illumination in comparison to treatment with either of the agents alone. In conclusion, simultaneous delivery and endo-lysosomal co-localization of Etop and PpIX by PAMAM(G5) Ns leads to a synergistic effect by phototherapy; in addition, the finding of antagonism without light illumination can be advantageous in lowering the dark toxicity and improving photo-selectivity.

## 1. Introduction

Glioblastoma multiforme (GBM) is the most common and aggressive primary malignant neoplasm of the central nervous system (CNS) in adults; it accounts for 14.7% of all primary CNS tumors and 56.6% of all gliomas [1]. GBM exhibits a predominant astrocytic differentiation and is classified by the World Health Organization Classification of Tumors of the Central Nervous System as grade IV astrocytoma [2]. The term “multiforme” refers to its variegated gross appearance and diverse histological features consisting of tumor cells with nuclear atypia or pleomorphism, high degrees of mitosis, microvascular proliferation, and necrosis. Further sub-classification is possible based on the specific alterations in gene expression of epidermal growth factor receptor, neurofibromatosis type I, platelet derived growth factor receptor and isocitrate dehydrogenase I into classical, mesenchymal, proneural, and neural subtypes. Each sub-type of GBM can be correlated with characteristic clinical presentation, response to treatment, and possible links to particular cells of origin, which highlights the heterogeneous nature of this pathologic entity [3]. Due to the diffusely infiltrative growth pattern of GBM, the prognosis of those affected is uniformly poor with an overall median survival of 6.1 months and a 5-year survival rate of less than 3.4%. The standard of care that consists of surgery, chemotherapy, and radiotherapy is only associated with a modest increase in survival, extending it by only a few months at best [4]. Furthermore, the treatment-related cost places substantial economic burden on the health care system. Thus, newer treatment strategies are needed.

Advances in fluorescence-guided surgery decades ago are one of the major breakthroughs in the management of GBM; the procedure involves the systemic administration of a photosensitizer (Ps) hours prior to anesthesia, which is preferentially taken into the tumor by the enhanced permeability and retention effect. Tumor resection is then carried out under a neurosurgical microscope, which illuminates the operative field with a wavelength that covers the absorption band of the Ps. Simultaneously, an optical filter which blocks the ambient and excitation light enables visualization of the fluorescence signal from the residual tumor real-time for surgical resection. The absence of fluorescence signal in the resection cavity ensures maximal resection, which is associated with a better disease-free and overall survival [5]. However, the full potential of such an approach from the broader perspective of phototherapy has yet to be realized. Phototherapy comprises of photodynamic therapy (PDT) and photochemical internalization (PCI). PDT utilizes tissue molecular oxygen, Ps, and light; these components are non-toxic on their own but when brought together can elicit photochemical reactions to generate cytotoxic reactive oxygen species (ROS) [6]. The preferential accumulation of Ps in tumor cells and localized light illumination allow dual selectivity for the temporo-spatial control of tissue destruction [7]. In PCI, a sub-lethal dose of Ps is used together with a therapeutic agent. The membrane-disruptive and membrane-permeabilization effects induced by photoactivated Ps can mediate endo-lysosomal escape of the co-administered therapeutic agent. Therefore, PCI not only enhances drug effect but also reduce drug toxicity. Furthermore, this approach may be advantageous when tumor hypoxia is encountered, because a lesser degree of the light-induced membrane-damaging effects is required for PCI to occur, which consumes less molecular oxygen than those needed for the full PDT effect [8,9,10]. Lastly, the fluorescence signal emitted from the relaxation of excited Ps to their ground state can also be used for photodiagnosis and aid in fluorescence-guided surgical resection of the targeted lesion as described before [11,12]. The Ps responsible for the fluorescence signal in GBM surgery is protoporphyrin IX (PpIX); it is a tetrapyrrole Ps containing 4 methyl, 2 propionic, and 2 vinyl side chains. PpIX is ubiquitously present in all living cells as a precursor of heme, cytochrome, and chlorophyll [13]. In mammals, 5-aminolevulinic acid (5-ALA) serves as a common precursor for all naturally occurring tetrapyrroles; it is derived from the condensation of glycine and succinyl-CoA in the mitochondria [14]. The excitation and emission wavelengths of PpIX is 409 nm and 633 nm, respectively. 5-ALA is administered as a source of endogenous PpIX for phototherapy [15], and more specifically for the fluorescence-guided surgery of GBM [16]. However, exogenous PpIX is not used alone because of its poor solubility and limited tumor selectivity [17].

The Stupp protocol consisting of radiotherapy plus concomitant and adjuvant chemotherapy with an alkylating agent (Temozolomide) has remained the standard of care for GBM since its publication in 2005 [18]. However, a combinatorial approach of adding a topoisomerase inhibitor to an alkylating agent has been proposed to address the issue of tumor heterogeneity and drug resistance for a lasting antitumor effect; one such example is etoposide (Etop) [19]. Etop is a semisynthetic derivative of podophyllotoxin; it is a cell-cycle-dependent antineoplastic drug that affects cells in the S and G2 phases of cell division. The formation of ternary complex with topoisomerase-II and DNA leads to DNA strand-breaks that prevent DNA repair and impair DNA synthesis [20]. This is followed by events leading to mitochondrial and caspase-dependent apoptosis and a dose-dependent G2/M cell cycle arrest [21,22]. Etop is effective against certain hematologic cancers, testicular tumors, and small cell lung cancer. Like most drugs, CNS Etop levels may be hindered by the blood–brain barrier; however, the brain tumor Etop concentration may range from 2% to 75% of the plasma concentration depending on the integrity of the blood–brain barrier [23]. Indeed, several studies utilizing Etop alone or in combination with other approved therapies have yielded significant gain of survival in patients with high grade glioma [24]. Etop, like many of the existing chemotherapeutic drugs, is poorly water-soluble and requires the inclusion of surfactants or co-solvents in the formulation that may be toxic or associated with adverse effects [25]. For this reason, encapsulation of these agents into nanodelivery systems has been suggested as an alternative to solvent-based solubilization approaches [26].

Nanodelivery systems have the advantage of optimizing drug loading and retention, improving circulation kinetics, tumor targeting, facilitating cellular uptake and intracellular drug release, thus enhancing therapeutic efficacy and safety [27]. The selection of nanodelivery system is based on its unique biochemical and biophysical properties towards the drug to be delivered and the site of intended action [28]. Dendrimers were chosen in this study to overcome the pharmacokinetic limitations imposed by the poor solubility of PpIX and Etop, and to co-localize these agents into the same subcellular compartment for maximal PCI [29]. Dendrimers belong to a class of nano-sized macromolecules, which has three components: a central core, an interior branching structure, and an exterior surface with functional groups [30]. The interior symmetric and layer-by-layer structure which consists of repeating units radially attached to the central core is referred to as generations. The branching configuration is well suited for drug encapsulation, whereas the surface can be modified with various functional moieties according to clinical need [30]. However, single dendrimers have limited drug delivery applicability owing to their small size, which range from 2.5 nm to 8 nm, which is just within the filtration-size threshold for renal clearance [31]. In general, particle size not exceeding 200 nm is considered ideal [32], therefore size-tunable core-shell structures such as dendrimicelles synthesized by the incorporation of amphiphilic block copolymers to the dendrimer-drug mixture or dendrimer aggregates (Nanospheres; Ns) formed by intermolecular interactions between dendrimer nanoparticles may be better suited for drug delivery [33,34].

**Scheme 1:**
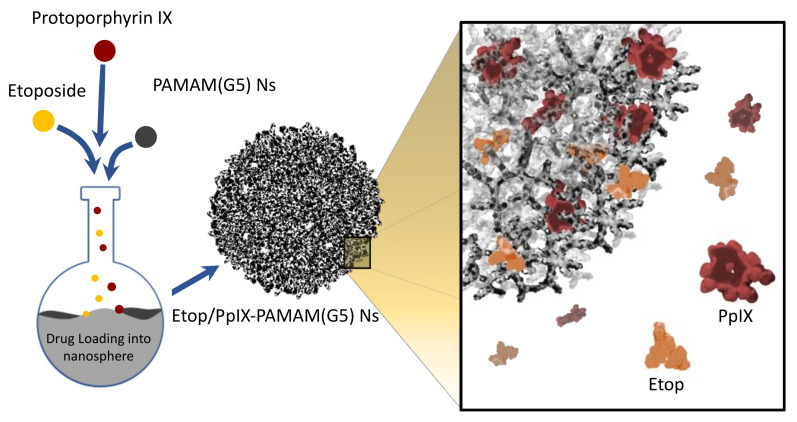
The Etop- and PpIX-loaded PAMAM(G5) Ns was synthesized by adding Etop and PpIX into pre-formed PAMAM(G5) Ns suspended in DMSO. For the purpose of PCI, a greater dose of Etop was used than PpIX. (DMSO—dimethyl sulfoxide, Etop—etoposide, PAMAM(G5) Ns—generation-5 polyamidoamine dendrimer nanosphere, PCI—photochemical internalization, PpIX—protoporphyrin IX).

In this study, dendrimer Ns were made from generation-5 polyamidoamine dendrimers (PAMAM(G5)) to carry Etop and a sub-lethal dose of PpIX (Etop/PpIX-PAMAM(G5) Ns) (Scheme 1). The confinement of both agents to one particle ensures subcellular co-localization necessary for effective PCI. The Ns were characterized and tested for phototherapy on a human GBM cell line by blue light illumination (409 nm) as an attempt to advance the treatment paradigm for GBM beyond the conventional scope of fluorescence-guided surgery. The intracellular localization and tumoricidal mechanism of phototherapy using the Etop/PpIX-PAMAM(G5) Ns were investigated in vitro.

## 2. Materials and Methods

### 2.1. Preparation of Etop/PpIX-PAMAM(G5) Ns

Generation-5 polyamidoamine dendrimers were purchased from Sigma-Aldrich (St. Louis, MO, USA). To generate the PAMAM(G5) Ns, 10 mg of PAMAM(G5) was dissolved in 5 mL of double-distilled water (ddH_2_O) and dropped in 2.5 mL of dimethyl sulfoxide (DMSO, Sigma-Aldrich). The mixture was stirred at 1150 rpm with a laboratory stirrer (PC-420D, Corning, Corning, NY, USA) at room temperature for 3 h. Centrifugation of the mixture was then performed through a centrifugal filter (Ultracel^®^, 10,000 MWCO, Millipore, Burlington, VT, USA) at 5250× *g* for 30 min in order to remove the DMSO. The product in the collection tube was re-suspended in 1 mL of ddH_2_O and the formation of Ns was confirmed by Zetasizer Nano ZS90 (Malvern, Worcestershire, UK). The sample in ddH_2_O containing PAMAM(G5) Ns was centrifugated through a centrifugal filter (Ultracel^®^, 10,000 MWCO, Millipore) at 5250× *g* for 30 min to isolate the PAMAM(G5) Ns, which was followed by re-suspension in 1 mL of DMSO for the drug loading steps below.

The method of drug loading into the PAMAM(G5) Ns was modified from those described by Shen et al. [35]; Etop and PpIX were loaded sequentially into the PAMAM(G5) Ns as follows: 2 mg of Etop (Sigma-Aldrich) in DMSO (25 µL from 136 mM stock) was dropped in the PAMAM(G5) Ns solution and stirred (1150 rpm, PC-420D, Corning) at room temperature for 1 h, and this was followed by the addition of 114 µg of PpIX (Sigma-Aldrich) in DMSO (16 µL from 10 mM stock), also stirred (1150 rpm) at room temperature for 1 h. The mixture was passed through a centrifugal filter at 5250× *g* for 30 min, and re-suspended in 1 mL of ddH_2_O before storage at 4 °C. All the procedures that involved handling of Ps were performed under dimmed light and the samples were shielded from ambient light exposure with tinfoil.

### 2.2. Characterization of PAMAM(G5) Ns and Etop/PpIX-PAMAM(G5) Ns

#### 2.2.1. Particle Size, Zeta Potential, Polydispersity Index, and Loading Efficiency of Etop/PpIX-PAMAM(G5) Ns

The Zetasizer Nano ZS90 (Malvern) was used to measure the cumulant Z-average diameter (Dav), zeta potential, and polydispersity index (PDI) of the Ns. The 1 mL sample containing the Ns in ddH_2_O was freeze-dried using SpeedVac DDA concentrator (Thermo Fisher Scientific, Waltham, MA, USA) for 24 h and then weighed. A 2 mg/mL sample of the Ns was prepared by suspension in ddH_2_O, then 0.5 mL of the sample was placed in a poly(methyl 2-methylpropenoate) semi-micro Vis cuvette (Eppendorf, Hamburg, Germany) at 25 °C for Dav and PDI measurements, and a 0.8 mL sample in a disposable capillary cell (DTS 1070, Malvern) was used for zeta potential measurement.

The loading efficiency of Etop and PpIX was determined by using 1 mL of Etop/PpIX-PAMAM(G5) Ns in DMSO through a centrifugal filter (Ultracel ^®^, Millipore) at 5250× *g* for 30 min. The amount of free Etop in the centrifuge tube was determined by a spectrophotometer (NanoDrop ND-1000; Thermo Fisher Scientific) on a 2 µL sample with the absorbance measurement set at 285 nm. Since only a trace amount of PpIX was used in the loading process, the amount of loaded PpIX was measured instead of the free PpIX. In order to facilitate dissolution of PpIX from the Etop/PpIX-PAMAM(G5) Ns for measurement, the Etop/PpIX-PAMAM(G5) Ns in the collection tube was re-suspended in 1 mL ddH_2_O and a 10 µL sample was added to 90 µL of DMSO and incubated at room temperature for 1 h. The amount of PpIX was determined using a spectrophotometer with the absorbance set at 405 nm. The loading efficiency was calculated as:

(1) $$\mathrm{Loading}\mathrm{Efficiency}\mathrm{Etop}\left(\%\right)=\frac{\left(\mathrm{Total}\mathrm{weight}\mathrm{of}\mathrm{Etop}-\mathrm{Weight}\mathrm{of}\mathrm{free}\mathrm{Etop}\right)}{\mathrm{Total}\mathrm{weight}\mathrm{of}\mathrm{Etop}}\times 100$$

(2) $$\mathrm{Loading}\mathrm{Efficiency}\mathrm{PpIX}\left(\%\right)=\frac{\mathrm{Weight}\mathrm{of}\mathrm{loaded}\mathrm{PpIX}}{\mathrm{Total}\mathrm{weight}\mathrm{of}\mathrm{PpIX}}\times 100$$

#### 2.2.2. Morphology and Structure of PAMAM(G5) Ns and Etop/PpIX-PAMAM(G5) Ns

The surface morphology of PAMAM(G5) Ns and Etop/PpIX-PAMAM(G5) Ns was obtained by a field emission scanning electron microscope (FE-SEM, SU8220, Tokyo, Japan). The samples on a conductive carbon paint in a specimen holder were vacuum-dried, and sputter-coated with platinum at 2 kV for 90 s. FE-SEM imaging of the sample was taken at 200,000× magnification. Transmission microsopy (TEM, H-7500, Hitachi, Tokyo, Japan) was performed also at 200,000× following negative staining with 2% (*w*/*v*) phosphotungstic acid (Sigma-Aldrich).

#### 2.2.3. Release Profile of Etop/PpIX-PAMAM(G5) Ns

The release profiles of Etop and PpIX from Etop/PpIX-PAMAM(G5) Ns were determined as follows: samples were prepared by passing the Etop/PpIX-PAMAM(G5) Ns in ddH_2_O (1 mL/tube) through a centrifugal filter at 5250× *g* for 30 min and re-suspended in of phosphate buffered saline (PBS). The 1 mL/tube samples in PBS were then incubated at 37 °C and 95% humidity. The samples from various time points were centrifugated at 18,000× *g*, 4 °C, and 30 min, and 2 µL of the supernatant was taken to measure the amounts of Etop and PpIX released using a spectrophotometer (NanoDrop ND-1000; Thermo Fisher Scientific) with the absorbance measurements set at 285 nm for Etop and 405 nm for PpIX. The release percentages were calculated by dividing the amounts of Etop and PpIX in the supernatant with the corresponding total amounts of Etop and PpIX loaded initially, which were taken from the loading efficiency experiments in the previous section.

### 2.3. In Vitro Dark and Photo-Cytotoxicities of Etop, PpIX, Etop Plus PpIX, and Etop/PpIX-PAMAM(G5) Ns, and the Analysis of Synergism by the Combination Index

The U87-MG human glioma cell line was obtained from the Bioresource Collection and Research Centre (Hsin Chu, Taiwan). The cells were maintained in Dulbecco’s modified Eagle’s medium (DMEM, Gibco, NY, USA) containing 10% fetal bovine serum (Gibco), 100 unit/mL penicillin G, and 100 g/mL streptomycin, and incubated under 5% CO_2_ at 37 °C. The cells were seeded onto a 96-well plate at a density of 6× 10^3^ cell/well and incubated overnight. The cells were treated for 18 h with different concentrations of Etop, PpIX, Etop plus PpIX, and Etop/PpIX-PAMAM(G5) Ns; the cells were then washed with PBS and replaced with fresh medium before light illumination (409 nm) for 0 min, 3 min, and 5 min. The 18 h incubation time was chosen to allow time for Etop to act on the cancer cells, which according to previous reports, ranges from 3 h to 48 h [36,37]. The 3-(4,5-Dimethylthiazol-2-yl)-2,5-diphenyltetrazolium bromide (MTT) assay was performed 48 h later. The dose increments of Etop/PpIX-PAMAM(G5) Ns were based on the equivalent doses of PpIX, which was extrapolated from the standard curve of fluorescence versus PpIX rather than the standard curve of fluorescence versus Etop/PpIX-PAMAM(G5) Ns. This was done in order to have a point of reference for comparison in between doses of PpIX and Etop/PpIX-PAMAM(G5) Ns. All the procedures that involved handling of Ps were performed under dimmed light and the well-plates were shielded from ambient light exposure with tinfoil. Light illumination was given to the specified duration by placing the well-plates on a custom-made illuminator (Bueno Optoelectronics Co. Ltd., Tainan, Taiwan); a flat-bed lamp containing four 10 Watt LT01-T82E-UV409 LED light tubes, which emitted blue light with a peak wavelength at 409 nm. The illuminator was air-cooled to prevent hyperthermia and provided homogeneous illumination over time with an output of 12 mW/cm^2^ (214.6 lumens), across a defined area of 30 × 50 cm. The illuminator was left switched on for at least 20 min to allow establishment of a stable irradiance before illumination of cells. 

Combination indices (CI) to assess synergy for Etop and PpIX combination or Etop/PpIX-PAMAM(G5) Ns were determined using CompuSyn v1.0 (ComboSyn, Inc., Paramus, NJ, USA), based on the Chou–Talalay method. 

### 2.4. In Vitro Cellular Uptake and Intracellular Localization of PpIX and Etop/PpIX-PAMAM(G5) Ns by U87-MG cells

The U87-MG cells were seeded at a density of 2 × 10^5^ cell/well on a 6-well culture dish and incubated in 5% CO_2_ at 95% humidity and 37 °C overnight. PpIX or Etop/PpIX-PAMAM(G5) Ns was added to the wells and cellular uptake was measured at different time points first by washing the wells with PBS, followed by trypsinization, centrifugation, and re-suspension in 0.2 mL PBS. The cells were passed through a polystyrene round-bottom tube with a cell-strainer cap (Corning) and intracellular PpIX was measured by a flow cytometer (FACScan, BD Biosciences, San Jose, CA, USA) with excitation set at 488 nm and emission set at 650 nm.

For intracellular localization of PpIX and Etop/PpIX-PAMAM(G5) Ns, 2 × 10^5^ cell/well of U87-MG cells were seeded on a 35 × 12 mm glass tissue culture dish and incubated overnight. The cells were treated with PpIX or Etop/PpIX-PAMAM(G5) Ns for 1 h and fluorescence images were taken by a confocal laser-scanning microscope (Leica TCS SP5II, Wetzlar, Germany) with the corresponding excitation and emission wavelengths for PpIX. 

The endo-lysosomal co-localization of PpIX and Etop/PpIX-PAMAM(G5) Ns was performed by first seeding 2 × 10^5^ cell/well of U87-MG cells on a 35 × 12 mm glass tissue culture dish and incubated overnight. The cells were treated with PpIX or Etop/PpIX-PAMAM(G5) Ns for 1 h followed by the addition of 50 nM LysoSensor™ Green DND-189 (Thermo Fisher Scientific) and incubated for 30 min before confocal microscopy. The excitation and emission wavelengths for LysoSensor™ Green DND-189 were set at 488 nm and 561 nm, respectively. The percentage of endo-lysosomal co-localization was determined using the Image Color Summarizer v0.76 by Martin Krzywinski (http://mkweb.bcgsc.ca/color/, accessed 4 November 2021); the confocal microscopy image files for PpIX fluorescence were uploaded, high precision was selected, and the number of color clusters were set at 5. The color cluster partition corresponding to the fluorescing PpIX inside the cells was selected, and the pixels representing all the intracellular PpIX was recorded. Similarly, the pixels representing PpIX or Etop/PpIX-PAMAM(G5) Ns co-localized to the endo-lysosome on the fused images were determined, then the proportion of co-localized color cluster pixels over those of all intracellular PpIX was calculated as a percentage.

### 2.5. Evaluation of Oxidative Stress and Apoptosis following Treatment of U87-MG Cells with Etop, PpIX PDT and Etop/PpIX-PAMAM(G5) Ns PCI

The oxidative stress of U87-MG cells following treatment with Etop, PpIX PDT, and Etop/PpIX-PAMAM(G5) Ns PCI was assessed by the dichloro-dihydro-fluorescein diacetate (DCFH-DA) assay using flow cytometry to detect the presence of the oxidized fluorescent product dichlorofluorescein (DCF). The level of DCFH-DA was assumed to be proportional to the concentration of ROS in the cells. The U87-MG cells were seeded at a density of 2 × 10^5^ cell/well on a 6-well culture dish and incubated overnight before treatment with Etop (0.9 µM), PpIX (0.3 µM), or Etop/PpIX-PAMAM(G5) Ns (containing 0.9 µM of Etop and 0.3 µM of PpIX) for 18 h. The doses of the respective agents were based on those of PpIX needed for PCI, which was associated with a sub-lethal PDT effect of approximately 30% to 50% cytotoxicity as determined from the previous experiments, and the corresponding doses of Etop/PpIX-PAMAM(G5) Ns and Etop were set accordingly. The cells were washed with PBS and replaced with fresh medium before light illumination for 0 min, 3 min, and 5 min, then incubated for 1.5 h before the addition of DCF (Sigma-Aldrich). After 30 min of incubation with DCF, the cells were washed with PBS, trypsinized, centrifugated, and re-suspended in 0.2 mL PBS with 250 pg propidium iodide (PI) (Sigma-Aldrich). Intracellular ROS was determined by the fluorescence signal of DCFH-DA in PI-positive cells using the flow cytometer.

Similarly, the Etop, PpIX, or Etop/PpIX-PAMAM(G5) Ns-treated cells were tested for apoptosis using the Annexin-V-FITC/PI apoptosis detection kit (BioLegend, San Diego, CA, USA). Following the respective treatments, the cells were washed, trypsinized, centrifugated, and re-suspended in 100 µL of binding buffer containing 3 × 10^6^ cells. Next, 5 µL of Annexin-V and 10 µL of PI were added and incubated for 15 min at room temperature in the dark, and then 400 µL of binding buffer was added before analysis by flow cytometry.

### 2.6. Statistical Analysis

The data are given as the mean with standard deviation. Statistical analyses were performed with the Stata version 11.0 statistical software (StataCorp LLC, College Station, TX, USA). The data were analyzed with two-way analysis of variance (ANOVA) and Tukey’s HSD test. A *p*-value of 0.05 or less was considered a statistically significant difference.

## 3. Results

### 3.1. Characterization of PAMAM(G5) Ns and Etop/PpIX-PAMAM(G5) Ns

The particle size, zeta potential, PDI, and loading efficiency of Etop and PpIX of the Ns are displayed in Table 1. The initial preparatory process utilizing PAMAM(G5) nanoparticles resulted in the creation of relatively well monodispersed larger-sized aggregates (PAMAM(G5) Ns) that measured 74.8 ± 1.1 nm in diameter; the second step by which Etop and PpIX were sequentially added led to the formation of nanoparticles (Etop/PpIX-PAMAM(G5) Ns) close to 3 times as large (217.4 ± 2.9 nm) and raised the PDI from 0.09 ± 0.01 to 0.24 ± 0.02. The corresponding loading efficiencies were 12% for Etop and 100% for PpIX. The zeta potential, which measured approximately 40 mV, remained more or less unchanged following the loading process, however.

**Figure 1:**
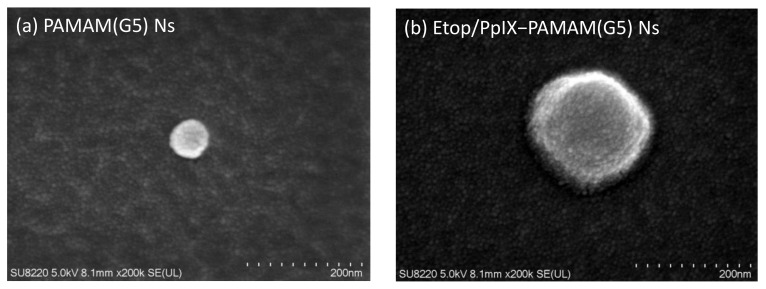
(**a**) FE-SEM image of PAMAM(G5) Ns; (**b**) Etop/PpIX-PAMAM(G5) Ns at 200,000× magnification. (Etop—etoposide, FE-SEM—field emission scanning electron microscope, PAMAM(G5) Ns—generation-5 polyamidoamine dendrimer nanosphere, PpIX—protoporphyrin IX).

On FE-SEM (Figure 1), both the PAMAM(G5) Ns and Etop/PpIX-PAMAM(G5) Ns appeared spherical in shape. The Etop/PpIX-PAMAM(G5) Ns exhibited a cobblestone surface texture which might indicate some degree of surface localization of Etop or PpIX.

The size difference was noticeable and comparable to measurements obtained from the Zetasizer Nano ZS90 (Figure 1).

**Figure 2:**
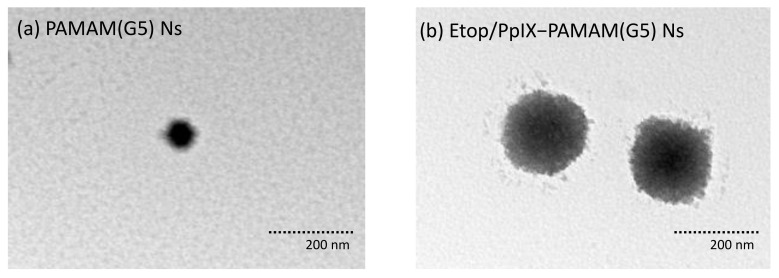
(**a**) TEM image of PAMAM(G5) Ns; (**b**) Etop/PpIX-PAMAM(G5) Ns at 200,000× magnification. (Etop—etoposide, PAMAM(G5) Ns—generation-5 polyamidoamine dendrimer nanosphere, PpIX—protoporphyrin IX, TEM—transmission electron micrograph).

Similar size and shape of the Ns were found on TEM (Figure 2); the PAMAM(G5) Ns appeared homogeneous in electron transparency, whereas the larger Etop/PpIX-PAMAM(G5) Ns had a denser core signifying its spherical morphology. 

### 3.2. Release Profile of Etop/PpIX-PAMAM(G5) Ns in Phosphate Buffered Saline by Absorbance Spectroscopy

**Figure 3:**
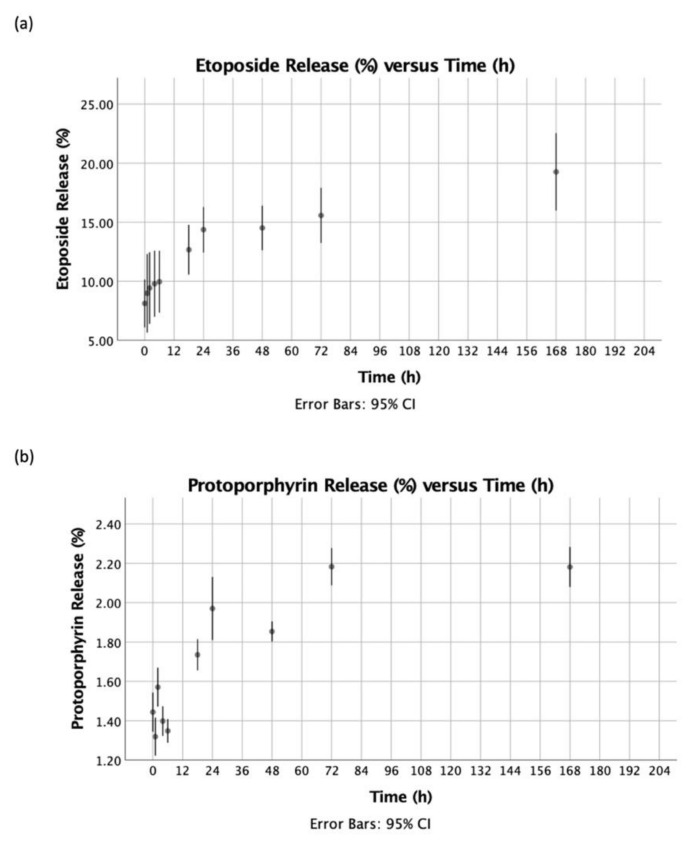
The release of Etop and PpIX from Etop/PpIX-PAMAM(G5) Ns was determined by absorbance spectroscopy. (**a**) The release profile of Etop; at time zero, 8.1 ± 3.0% of free Etop appeared to exist with the Etop/PpIX-PAMAM(G5) Ns. The release of Etop was observed up to 3 days (15.6 ± 3.5%), which then stopped from days 3 to 7. Only 19.3 ± 4.6% of free etoposide was found at day 7. (**b**) The release profile of PpIX; at time zero, 1.4 ± 0.2% of free PpIX was detected, which also rose within the first 3 days (2.2 ± 0.1%), and stopped from days 3 to 7. The percentage of free PpIX at day 7 remained unchanged. *n* = 8. (Etop—etoposide, PAMAM(G5) Ns—generation-5 polyamidoamine dendrimer nanosphere, PpIX—protoporphyrin IX).

The release profile of Etop and PpIX from the Etop/PpIX-PAMAM(G5) Ns in PBS taken at different time points was determined by absorbance spectroscopy. The result was expressed as a percentage of the free to total amount of Etop and PpIX (Figure 3a,b). At time zero, 8.1 ± 3.0% of free Etop appeared to exists with the Etop/PpIX-PAMAM(G5) Ns. The release of Etop rose to 15.6 ± 3.5% by day 3, but no further release was seen from days 3 to 7 (19.3 ± 4.6%) when taking account of the standard deviations. Similarly, 1.4 ± 0.2% of free PpIX was present at time zero and the release over time was minute which essentially stopped by day 3 (2.2 ± 0.1%). The percentage of PpIX released by day 7 remained unchanged. The release profile suggests the establishment by day 3 of an equilibrium between the free and the Ns-loaded agents in the experimental system.

### 3.3. In Vitro Dark and Photo-Cytotoxicities of Etop, PpIX, Etop plus PpIX, and Etop/PpIX-PAMAM(G5) Ns against U87-MG Cells, and the Analysis of Synergism by the Combination Index

**Figure 4:**
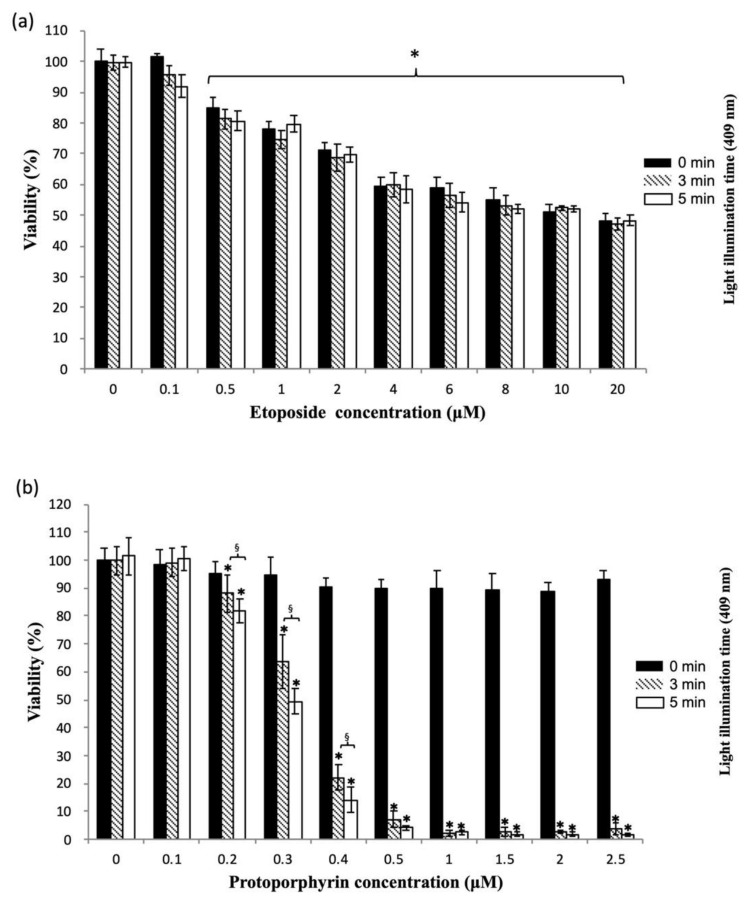

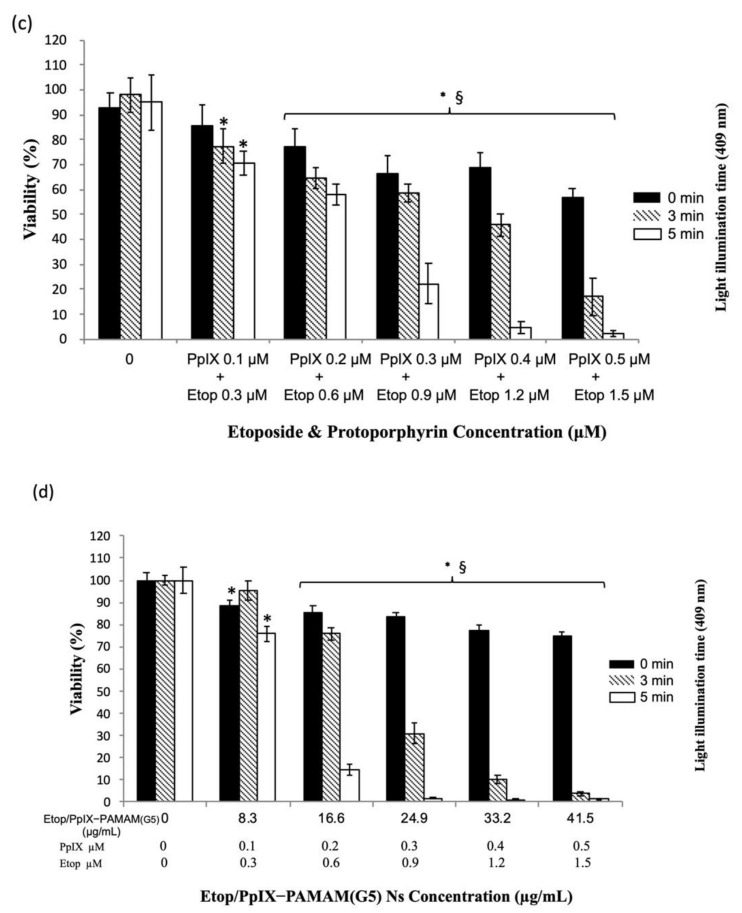
The MTT assay was performed 48 h after treatment of U87-MG cells with different drug concentrations and light illumination times. (**a**) The effect of Etop appeared to abate with dose escalation towards 20 µM, reaching a maximal cytotoxicity of approximately 50%. (**b**) PDT with PpIX alone lead to significant dose-dependent cytotoxicity, reaching greater than 95% cytotoxicity from 0.5 µM of PpIX and beyond. Illumination-time dependence was observed from 0.2 µM to 0.4 µM, whereas dark toxicity was not encountered in the concentration range tested (0.1 µM to 2.5 µM). (**c**) Phototherapy using co-administered Etop and PpIX led to significant cytotoxicity which was both dose- and illumination time-dependent. (**d**) Phototherapy using Etop/PpIX-PAMAM(G5) Ns also revealed significant effects which were both dose- and illumination-time dependent. * *p* < 0.05 (versus control). § *p* < 0.05 (same dose of Etop and PpIX or Etop/PpIX-PAMAM(G5) Ns versus different illumination times). *n* = 6. (Etop—etoposide, PAMAM(G5) Ns—generation-5 polyamidoamine dendrimer nanosphere, PpIX—protoporphyrin IX).

The dose-escalating cytotoxic effects on U87-MG cells of Etop, PpIX PDT, phototherapy by Etop/PpIX combination and Etop/PpIX-PAMAM(G5) Ns determined by the MTT assay are shown in Figure 4. Light illumination in phototherapy was given for 3 and 5 min. Treatment with Etop led to a dose-dependent reduction in tumor cell viability that began at a drug concentration of 0.5 µM and plateaued to approximately 50% at a drug concentration of up to 20 µM; light illumination showed no significant in vitro tumoricidal activity, thus confirming the lack of photosensitizing effect on Etop (Figure 4a). No dark toxicity was induced by PpIX at concentrations of 0.1 µM to 2.5 µM. PDT-induced cytotoxic effect began at 12 ± 6.8% with 0.2 µM of PpIX and 3 min light illumination, which reached in excess of 95% cytotoxicity from 0.5 µM of PpIX and beyond, and extending the light illumination time to 5 min led to significant differences in cytotoxicity at lower PpIX concentrations, but no added benefit was observed at 0.5 µM or more of PpIX (Figure 4b). The effect of phototherapy by increasing the dose of co-administered Etop and PpIX at a fixed ratio of 3:1, from 0.3 µM Etop, 0.1 µM PpIX to 1.5 µM Etop, 0.5 µM PpIX is shown in Figure 4c; a significant increase in the in vitro tumoricidal activity was observed from 23 ± 6.9% to 83 ± 7.5% with 3 min light illumination, and from 30 ± 5.0% to 98 ± 1.2% with 5 min light illumination. Significant cytotoxicity in the absence of light illumination was noted at 0.6 µM Etop, 0.2 µM PpIX to 1.5 µM Etop, 0.5 µM PpIX, which rose from 23 ± 7.4% to 43 ± 3.9%. Finally, the treatment effect of Etop/PpIX-PAMAM(G5) Ns is represented in Figure 4d, the doses of the Ns were chosen to contain an equivalent amount of Etop and PpIX at a fixed ratio of 3:1; significant dose-dependent and illumination-time dependent in vitro tumoricidal effects were observed at a Ns concentration of 8.3 µg/mL (equivalent of Etop 0.3 µM and PpIX 0.1 µM) to 41.5 µg/mL (equivalent of Etop 1.5 µM and PpIX 0.5 µM); the in vitro tumoricidal effect increased from 12 ± 3.0% to 25 ± 2.4% without light illumination, from 5 ± 4.2% to 96 ± 0.8% with 3 min light illumination, and from 24 ± 3.6% to 99 ± 0.5% with 5 min light illumination.

**Figure 5:**
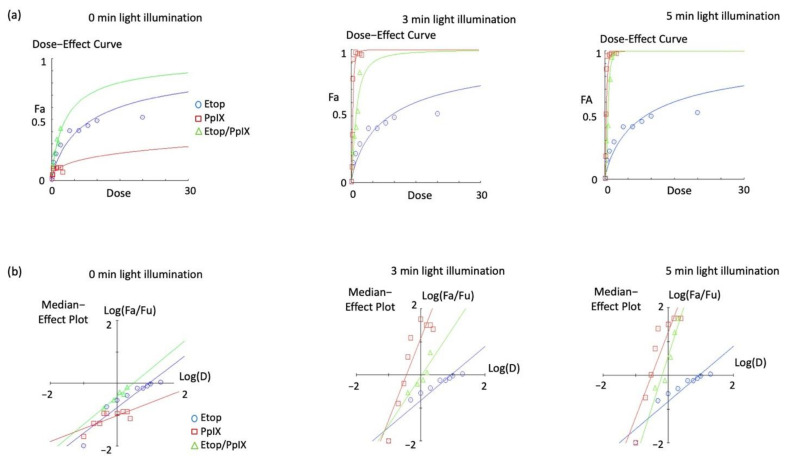

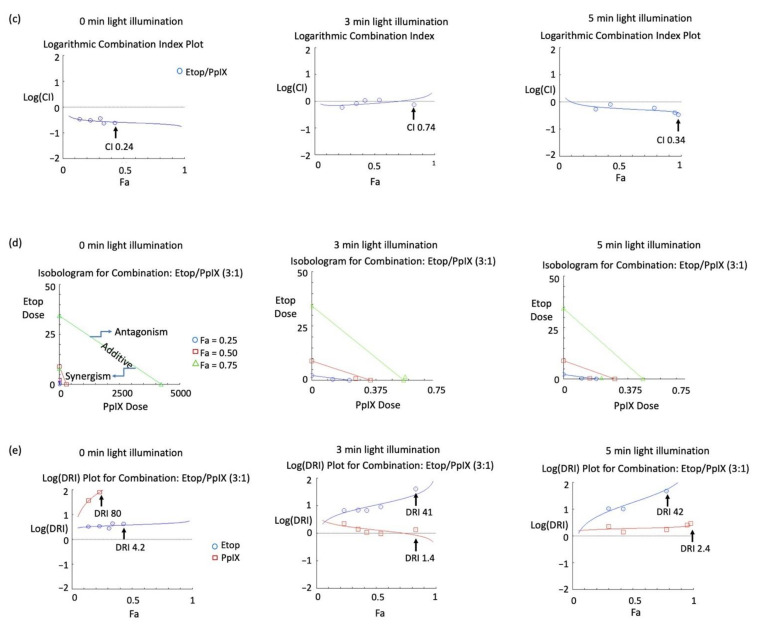
The graphical outputs from the CompuSyn software using cytotoxicity data for Etop, PpIX PDT, and phototherapy with Etop plus PpIX. (**a**) The dose-effect curve showed superior response for Etop plus PpIX even in the absence of light illumination; the response profile was below that of PpIX PDT for 3 min light illumination and became almost identical to that of PpIX PDT at 5 min light illumination. (**b**) The light-dependent effect of PpIX PDT and phototherapy with Etop plus PpIX was manifested by a shift in the x-intercept on the median-effect plot. (**c**) The CI at the highest tested dose revealed strong synergism for Etop plus PpIX without light illumination, synergism for 5 min light illumination, and moderate synergism for 3 min light illumination. (**d**) The isobologram showed regions of synergism and antagonism for specific Fa; an illumination time-dependent dose reduction possible for both Etop and PpIX in the combined treatment was evident. (**e**) Phototherapy with Etop plus PpIX was associated with a favorable dose reduction for Etop and less so for PpIX at the high Fa range. Dose reduction was also possible in the absence of light illumination, but this was less relevant due to the low attainable Fa value in the tested dose range. (CI—combination index, DRI—dose-reduction index, Etop—etoposide, PDT—photodynamic therapy, PpIX—protoporphyrin IX).

**Figure 6:**
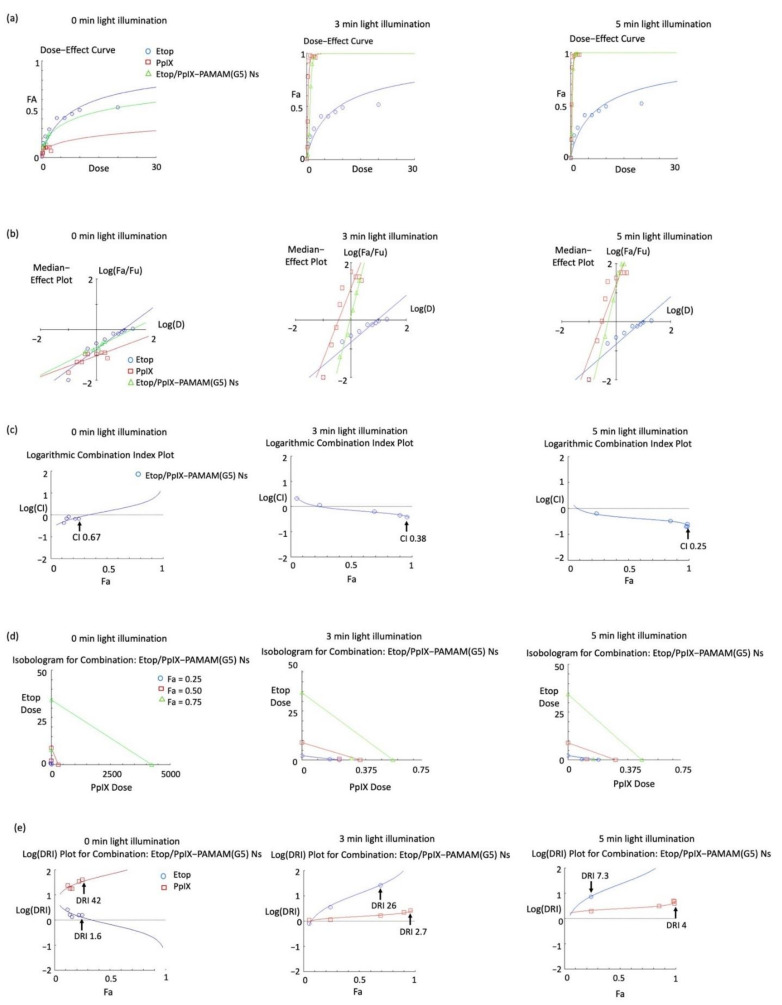
The graphical outputs from the CompuSyn software using cytotoxicity data for Etop, PpIX PDT, and Etop/PpIX-PAMAM(G5) Ns phototherapy. (**a**) Incorporation of Etop and PpIX into the Ns showed mixed effects when compared with Etop alone, the dose effect ranged from synergism in the low dose range to antagonism in the high dose range in the absence of light illumination; 3 min light illumination resulted in an earlier climb to plateau than Etop plus PpIX. (**b**) The light-dependent effect PpIX PDT and Etop/PpIX-PAMAM(G5) Ns phototherapy was manifested by a shift in the x-intercept on the median effect plot with PpIX PDT being more potent than Etop/PpIX-PAMAM(G5) Ns phototherapy. (**c**) Synergism was observed with 0 min of light illumination but only in the low Fa range, whereas synergism and strong synergism were seen with 3 min and 5 min light illumination in the high Fa range, respectively. (**d**,**e**) The isobologram appeared very similar to phototherapy with Etop plus PpIX in which a favorable dose reduction was possible in the high Fa range especially for Etop and less so for PpIX. (CI—combination index, DRI—dose-reduction index, Etop—etoposide, PAMAM(G5) Ns—generation-5 polyamidoamine dendrimer nanosphere, PDT—photodynamic therapy, PpIX—protoporphyrin IX).

Data derived from the cytotoxicity experiments above for Etop, PpIX PDT, phototherapy with Etop/PpIX combination, and Etop/PpIX-PAMAM(G5) Ns were subjected to a computerized analysis of synergism or antagonism by the Chou–Talalay method on the CompuSyn software [38]. The graphical outputs are displayed for phototherapy using Etop/PpIX combination (Figure 5) and Etop/PpIX-PAMAM(G5) Ns (Figure 6); these include (a) dose effect, (b) median effect, (c) logarithmic combination index (LogCI), (d) isobologram and (d) logarithmic dose-reduction index (LogDRI) for drug combination. CIs of <1, =1, and >1 indicates synergism, additive, and antagonism respectively, and a semi-quantitative expression was used for descriptive purposes [39]. A dose-reduction index (DRI) of >1, =1, and <1 indicates favorable, no dose reduction, and unfavorable dose reduction, respectively. The doses for Etop/PpIX combination and Etop/PpIX-PAMAM(G5) Ns were entered as the sum of Etop and PpIX, at a ratio of 3:1; thus, this contained only 3/4 and 1/4 the dose of Etop and PpIX in comparison to the dose effect of free Etop and PpIX.

The dose effect curve shows the cytotoxicity of the respective agents; notably, the tracing for Etop/PpIX combination without light illumination was higher than either agent alone, while 3 min light illumination resulted in an intermediate profile just below that of PpIX PDT and an earlier climb to plateau as light illumination was extended to 5 min (Figure 5a). The relative potencies of the agents are represented by the x-intercept on the median effect plot. Understandably, the light-dependent effect of phototherapy was manifested by the increase in the potencies of PpIX and Etop/PpIX combination (Figure 5b). Synergism for drug combination is more desirable in the high Fa (effect) range for anti-cancer therapy. The CI for Etop/PpIX phototherapy at the highest tested dose showed synergism for 5 min light illumination, moderate synergism for 3 min light illumination, and strong synergism without light illumination (Figure 5c). The isobologram identifies region of dose combination separated by the diagonal line (line of additive effect) for specific Fa value, below which indicates synergism and above which indicates antagonism; the plot indicated a greater degree of dose reduction possible for Etop than PpIX in the Etop/PpIX combination to maintain a synergistic effect by phototherapy (Figure 5d). Dose reduction for the respective agents was demonstrated more clearly in the LogDRI plot as an illumination time-dependent favorable dose reduction in the high Fa range; similarly, dose reduction was possible in the absence of light illumination but this was less relevant due to the low attainable Fa value in the tested dose range (Figure 5e).

The dose effect tracing for Etop/PpIX-PAMAM(G5) Ns without light illumination showed a marginally higher cytotoxicity than that of Etop alone at low doses then became lower as the dose was increased (Figure 6a). An earlier climb to plateau with Etop/PpIX-PAMAM(G5) Ns in the dose effect tracing than combined therapy of Etop plus PpIX with 3 min light illumination was evident. A light illumination time-dependent increase in potency was also demonstrated for which PpIX was again the most potent (Figure 6b). The shift of CI between Etop and PpIX without light illumination was better appreciated in the LogCI plot; synergism was observed with 0 min of light illumination but only in the low Fa range, whereas synergism and strong synergism in the high Fa range were seen with 3 min and 5 min light illumination, respectively (Figure 6c). The isobologram appeared very similar to Etop plus PpIX (Figure 5d) with a favorable dose reduction possible in the high Fa range especially for Etop and less so for PpIX (Figure 6c).

### 3.4. In Vitro U87-MG Cellular Uptake of PpIX and Etop/PpIX-PAMAM(G5) Ns by Flow Cytometry, and Intracellular Localization by Confocal Microscopy

**Figure 7:**
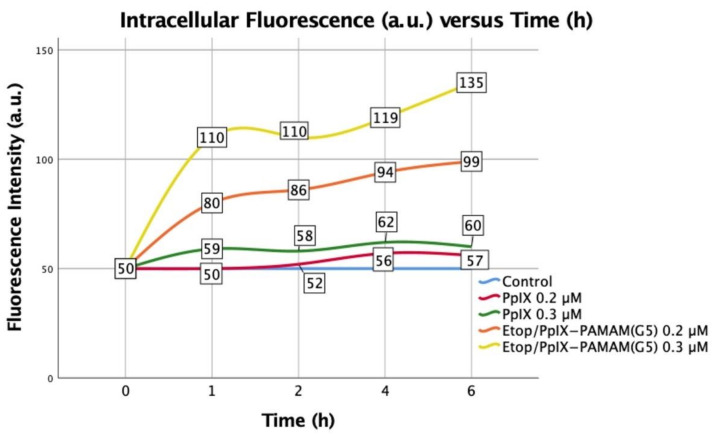
The U87-MG cellular uptake of PpIX and Etop/PpIX-PAMAM(G5) Ns was determined by the fluorescence intensity of PpIX on flow cytometry. The baseline autofluorescence from time 0 h to 6 h was measured at 50 a.u. The uptake with 0.2 µM of PpIX was slow and minimal, but this was increased when the dose was raised to 0.3 µM. The uptake of Etop/PpIX-PAMAM(G5) Ns was nearly two times the corresponding dose of PpIX, which appeared rapid within the first hour then more gradually thereafter. (Etop—etoposide, PAMAM(G5) Ns—generation-5 polyamidoamine dendrimer nanosphere, PpIX—protoporphyrin IX).

The U87-MG cellular uptake of PpIX and Etop/PpIX-PAMAM(G5) Ns were evaluated by flow cytometry at various time points following administration. The cellular uptake was quantified by the fluorescence intensity of PpIX, which was plotted against time as shown in Figure 7. The doses of PpIX chosen either in their free form or incorporated in the Ns (0.2 µM and 0.3 µM equivalence) were based on those best suited for PCI, which were doses associated with a sub-lethal PDT effect from the previous section. The cellular uptake using 0.2 µM of PpIX was minimal but steady. Although this was increased by raising the dose to 0.3 µM, the uptake remained low. The Etop/PpIX-PAMAM(G5) Ns was associated with an almost twofold rise in cellular uptake which was also dose-dependent. The pattern of increase was rapid within the first hour and then became more gradually as time progressed.

**Figure 8:**
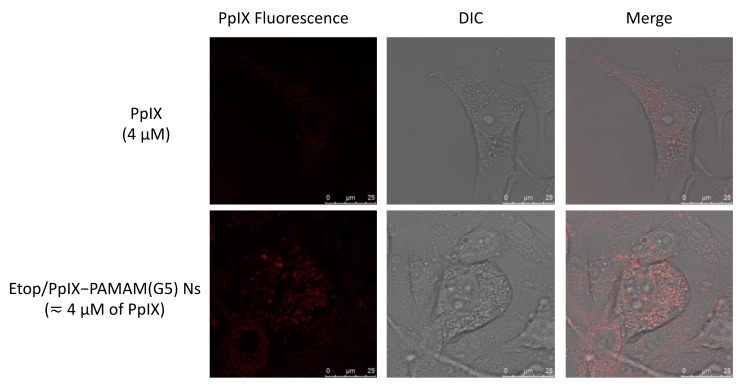
U87-MG cells incubated with PpIX or Etop/PpIX-PAMAM(G5) Ns for 1 h were imaged by confocal microscopy; fluorescence images were taken with the excitation and emission wavelengths for PpIX which were 409 nm and 570 nm, respectively. DIC microscopy images were taken concurrently for image fusion. PpIX fluoresces red on confocal microscopy upon excitation. The intracellular fluorescence was much more evident for Etop/PpIX-PAMAM(G5) Ns than for PpIX, and these appeared to localize as dense granules within the cytoplasm. (DIC—differential interference contrast, Etop—etoposide, PAMAM(G5) Ns—generation-5 polyamidoamine dendrimer nanosphere, PpIX—protoporphyrin IX). The scale bars = 25 µm.

Confocal microscopy images of U87-MG cells following incubation with PpIX or Etop/PpIX-PAMAM(G5) Ns for 1 h are shown in Figure 8; the red fluorescence signal of PpIX indicated cellular uptake and appeared to congregate in small granules in the cytoplasm; the intracellular fluorescence signal intensity appeared much higher for Etop/PpIX-PAMAM(G5) Ns than for PpIX, which was in agreement with the fluorescence intensity data obtained from flow cytometry.

**Figure 9:**
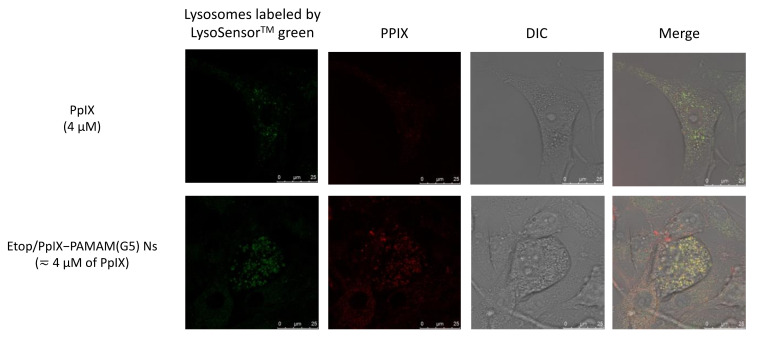
U87-MG cells incubated with PpIX or Etop/PpIX-PAMAM(G5) Ns for 1 h and then with LysoSensor™ Green for 30 min were imaged by confocal microscopy; fluorescence images were taken with the excitation and emission wavelength for PpIX (409 nm/570 nm) and LysoSensor™ Green (488 nm/561 nm). DIC microscopy images were taken for image fusion. PpIX fluoresces red while lysosomes labeled by LysoSensor™ Green fluoresces green. PpIX, Etop/PpIX-PAMAM(G5) Ns, and LysoSensor™ Green appeared to co-localize on the merge image, which indicated that these agents were in the endo-lysosomal compartment. (DIC—differential interference contrast, Etop—etoposide, PAMAM(G5) Ns—generation-5 polyamidoamine dendrimer nanosphere, PpIX—protoporphyrin IX). The scale bars = 25 µm.

The subcellular localization of PpIX and Etop/PpIX-PAMAM(G5) Ns were evaluated by confocal microscopy with concurrent lysosome labeling by LysoSensor™ Green (Figure 9); the red fluorescence signal from the PpIX and the green fluorescence signal from the LysoSensor™ Green appeared to be co-localized in the endo-lysosomal compartment as indicated by the yellowish color when both images were merged. The percentages of endo-lysosomal co-localization were calculated as 51.8 ± 8.3% for PpIX and 76.8 ± 3.0% for Etop/PpIX-PAMAM(G5) Ns, and the differences were statistically significant.

### 3.5. Evaluation of Oxidative Stress by the Dichloro-Dihydro-Fluorescein Diacetate Assay and Apoptosis by the Annexin V-FITC/PI Apoptosis Detection Kit following Treatment of U87-MG Cells with Etop, PpIX PDT, and Etop/PpIX-PAMAM(G5) Ns PCI

**Figure 10:**
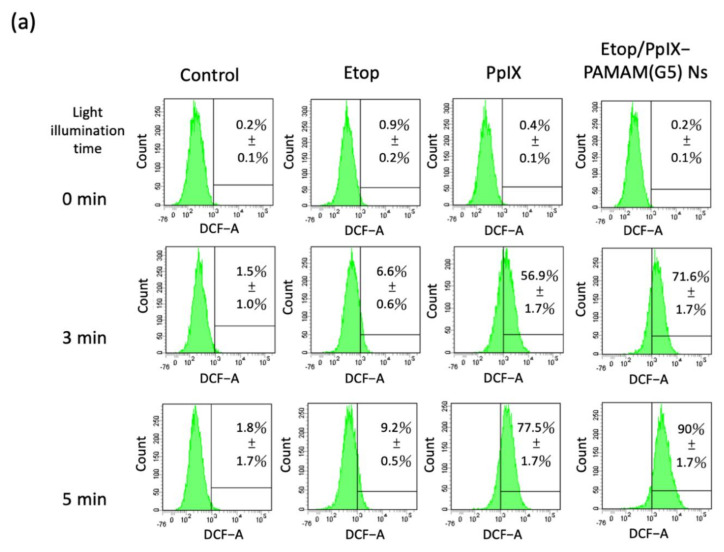

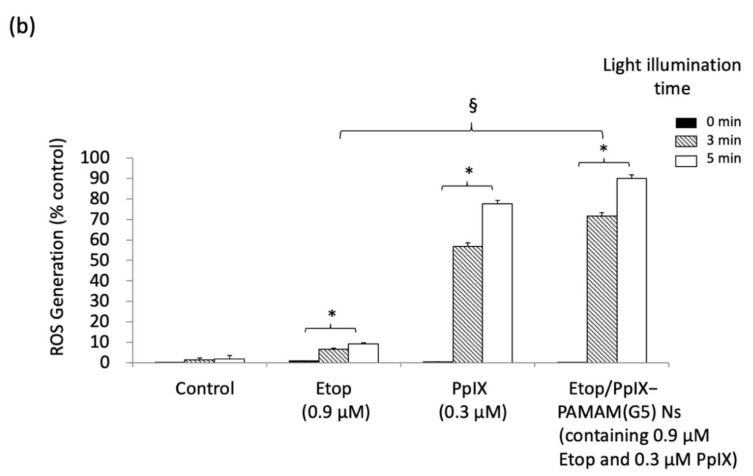
The generation of intracellular ROS in U87-MG cells following treatment with Etop (0.9 µM), PpIX (0.3 µM) PDT, and Etop/PpIX-PAMAM(G5) Ns (containing 0.9 µM Etop and 0.3 µM PpIX) PCI were analyzed using flow cytometry of oxidized DCFH-DA; the oxidized fluorescent product DCF corresponds to the redox state of the cells, which is assumed to be proportional to the concentration of ROS in the cells. (**a**) The single parameter histogram showed the percentage of cells with DCF fluorescence with varying treatment; the percentages ranged from 0.87% to 9.2% for Etop, 0.37% to 77.53% for PpIX PDT, and 0.23% to 90% for Etop/PpIX-PAMAM(G5) Ns PCI from 0 min to 5 min light illumination. (**b**) Significant increases in the proportion of cells bearing DCF fluorescence were observed across all treatments following light illumination; these were positively correlated to the light illumination time with Etop/PpIX-PAMAM(G5) Ns PCI showing significantly higher percentages than all other treatments. * *p* < 0.05 (differences within treatment group). § *p* < 0.05 (differences in between treatment groups using the same light illumination time). (DCF—dichlorofluorescein, DCFH-DA—dichloro-dihydro-fluorescein diacetate, Etop—etoposide, PAMAM(G5) Ns—generation-5 polyamidoamine dendrimer nanosphere, PCI—photochemical internalization, PDT—photodynamic therapy, PpIX—protoporphyrin IX, ROS—reactive oxygen species).

Oxidative stress of the U87-MG cells following treatment with Etop, PpIX PDT, and Etop/PpIX-PAMAM(G5) Ns PCI was determined by the DCFH-DA assay using flow cytometry. The percentages of cells that showed DCF fluorescence with varying treatment are displayed as single parameter histograms in Figure 10a and as a bar graph for comparison in Figure 10b; the proportion of cells bearing DCF fluorescence was low and not significantly different between Etop, PpIX, and Etop/PpIX-PAMAM(G5) Ns in the dark. However, an illumination time-dependent rise was observed across all treatments, which increased from 0.87% to 9.2% for Etop, 0.37% to 77.53% for PpIX PDT, and 0.23% to 90% for Etop/PpIX-PAMAM(G5) Ns PCI from 0 min to 5 min light illumination with Etop/PpIX-PAMAM(G5) Ns PCI showing significantly higher percentages than all other treatments.

**Figure 11:**
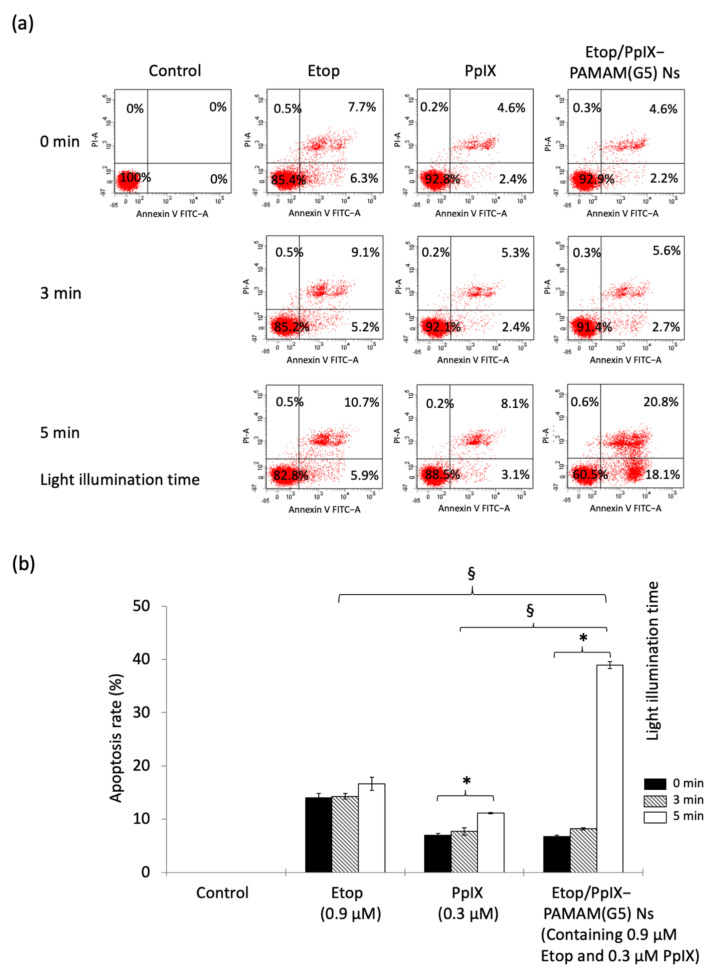
Apoptosis of U87-MG cells following treatment with Etop (0.9 µM), PpIX (0.3 µM) PDT, and Etop/PpIX-PAMAM(G5) Ns (containing 0.9 µM Etop and 0.3 µM PpIX) PCI was evaluated using the Annexin V-FITC/PI apoptosis detection kit. (**a**) The two-parameter dot plots from flow cytometry using Annexin V probe conjugated to FITC and PI distinguished cells of early (right lower quadrant) and late (right upper quadrant) apoptosis, respectively. (**b**) The sum of Annexin V-FITC and PI-positive cells were taken as the total proportion of apoptotic cells, which was 14.3% for Etop, 7% for PpIX, and 6.8% for Etop/PpIX-PAMAM(G5) Ns at baseline. Light illumination did not change the percentage of apoptosis for Etop significantly, and only slightly for 3 min and 5 min PpIX PDT and 3 min Etop/PpIX-PAMAM(G5) Ns PCI, whereas a marked increase was observed for 5 min Etop/PpIX-PAMAM(G5) Ns PCI. Apoptosis following Etop treatment was significantly higher than 3 min and 5 min PpIX PDT and 3 min Etop/PpIX-PAMAM(G5) Ns PCI. * *p* < 0.05 (differences within treatment group). § *p* < 0.05 (differences in between treatment groups using the same light illumination time). (Etop—etoposide, PAMAM(G5) Ns—generation-5 polyamidoamine dendrimer nanosphere, PCI—photochemical internalization, PDT—photodynamic therapy, PI—propidium iodide, PpIX—protoporphyrin IX).

An Annexin V-FITC/PI apoptosis detection kit was used to evaluate the mechanism of cell death following treatment with Etop, PpIX PDT, and Etop/PpIX-PAMAM(G5) Ns PCI. U87-MG cells at different apoptotic periods were distinguished by flow cytometry using Annexin V probe conjugated to FITC and PI as markers of early and late apoptosis, respectively, and the resultant two parameter dot-plots are represented in Figure 11a. The sum of Annexin V-FITC and PI-positive cells were taken as the total proportion of apoptotic cells which are shown in Figure 11b. Apoptosis was associated with all agents which measured 14.3% for Etop, 7% for PpIX, and 6.8% for Etop/PpIX-PAMAM(G5) Ns at baseline. PpIX PDT only led to a small rise in the percentage of apoptosis to 7.7% and 11.2% with 3 min and 5 min light illumination, respectively, whereas the increase was up to 38.9% for Etop/PpIX-PAMAM(G5) Ns PCI. Apoptosis following Etop treatment was significantly higher than 3 min and 5 min PpIX PDT and 3 min Etop/PpIX-PAMAM(G5) Ns PCI, but was significantly lower than 5 min Etop/PpIX-PAMAM(G5) Ns PCI.

## 4. Discussion

Photochemical internalization is a strategy for endo-lysosomal escape of drugs at the subcellular level that allows enhanced drug efficacy, reduced drug adverse effects and skin photosensitivity, and improved drug selectivity. PCI protocol is a stepwise process that usually involves the systemic administration of a Ps followed by a chemotherapeutic agent and then activation by light, in the so-called “light after” drug regime [40]. The subcellular localization of Ps impacts on the effectiveness of phototherapy and varies according to the mechanism of cellular uptake, which may include diffusion, passive partitioning, endocytosis, and pinocytosis [41,42,43]. For example, the first generation Ps such as hematoporphyrin derivative localizes diffusely in the cytomembrane and porfimer (Photofrin^®^) localizes to the Golgi apparatus and the endoplasmic reticulum [44,45], while the second generation Ps such as *meta*-tetrahydroxy-phenylchlorin (Foscan^®^), 5-ALA, and N-aspartyl chlorin e6 accumulates in the Golgi apparatus and endoplasmic reticulum, mitochondria, and lysosome, respectively [46,47,48]. In order for effective PCI to occur, the preferential uptake by endocytosis and accumulation of Ps in the endo-lysosomal axis is a crucial requisite [49,50]; these can be mediated by the conjugation of cell penetrating peptide or low density lipoprotein to the Ps, or through the use of Ps in the form of nanocomposites or nanoparticles [51,52,53]. In this study, the latter approach was utilized through the formation of PAMAM(G5) Ns incorporating both Etop and PpIX. The relative doses of Etop and PpIX for loading into the PAMAM(G5) Ns were specifically chosen for PCI application in which Etop was the therapeutic agent of interest, and the PpIX provided the necessary photochemical reaction in the release mechanism without exerting toxicity. Accordingly, the resultant Etop/PpIX-PAMAM(G5) Ns contained approximately twice the amount of Etop than PpIX by calculation based on the respective loading efficiencies. The utilization of PAMAM(G5) Ns not only overcame the issue of aqueous solubility of the active ingredients but also enabled organelle-targeting of Etop and PpIX to the endo-lysosomal compartment as indicated by the fluorescence signal of PpIX in the Ns on confocal microscopy. The cellular uptake experiments by flow cytometry not only revealed that the cells were able to internalize the Etop/PpIX-PAMAM(G5) Ns within hours of treatment, but it also showed an almost twofold increase in the associated amount of intracelllar PpIX when compared with treatment by free PpIX. The favorable uptake characteristics could potentially shorten the waiting time in between drug administration and light illumination. Although 5-ALA is approved for clinical use, the selection of 5-ALA for the purpose of PCI in this study is less well suited, mainly because of the metabolic steps necessary to convert the prodrug form into PpIX and the predominant redistribution of PpIX into the mitochondria, which is ineffective for PCI of the co-administered therapeutic agent.

Multi-drug regimens intended for therapeutic synergy are common in cancer therapy and the use of nanoparticles as a delivery platform ensures the stability, targeted delivery, and controlled release of chemically dissimilar drugs [54]. PAMAM(G5) Ns was used in this study as the delivery platform; an increase in size of the Ns following loading with Etop and PpIX was observed, which is most likely to be related to further agglomeration of the pre-formed PAMAM(G5) Ns associated with the interchanging protic and aprotic solvent systems needed in the synthetic process. The unchanged surface charge and the extended release-profile of the Etop/PpIX-PAMAM(G5) Ns probably indicate loading of the non-charge bearing and comparatively small-by-weight molecules of Etop and PpIX into the interior branching structure of dendrimers. However, the cobblestone-like surface feature on the Etop/PpIX-PAMAM(G5) Ns by FE-SEM might suggest some degree of surface localization of Etop or PpIX through non-covalent interactions. The additional benefit of enhanced cellular uptake mediated by the Ns was also observed and could be partly responsible for the greater in vitro tumoricidal effect by phototherapy than by the combination of free Etop and PpIX. To further explore the possibility of synergism between Etop and PpIX phototherapy, the Chou–Talaly method was used to provide a quantitative measure for the drug combinations. This is a simple and versatile method based on the law of mass-action which is mechanism-independent and applicable in both in vitro and in vivo settings [55]. Furthermore, the extension of this method to nanoparticles for combination drug therapy has been described in other studies [56,57,58,59]. 

Synergism for the Etop and PpIX combination given in the form of free drugs or within PAMAM(G5) Ns could be explained by several mechanisms in addition to PCI. These might include altered drug-protein binding, enhanced cellular drug uptake and evasion of efflux mechanism, and oxidative activation of Etop. The strong synergism of Etop plus PpIX without light illumination could be related to the extensive and reversible protein binding of both Etop and PpIX in the biologic medium [60,61]. Accordingly, some degree of competitive protein binding is expected when these agents are given together. Therefore, protein binding displacement by PpIX, which is minimally toxic in the dark, could potentially increase the cytotoxicity from the action of unbound Etop. However, synergism in this setting is undesirable because it limits tumor and photo-selectivity even though the Fa range was below 0.5. Oxidative activation of Etop with enhanced anti-cancer effects has been described to comprise a direct and an indirect mechanism [62]. The direct mechanism relates to its unique chemical structure, the radical-scavenging pendant phenolic E-ring [63] which is converted to phenoxyl radicals by the donation of hydrogen to peroxyl radicals generated from intracellular processes such as enzymatic oxidative metabolism or oxidative stress [64]. The phenoxyl radicals not only deplete the endogenous intracellular anti-oxidant glutathione, but also oxidize critical protein thiols to cause an increase in intracellular oxidative stress and oxidative DNA damage in addition to DNA strand cleavage [65]. The indirect mechanism involves the depletion of intracellular anti-oxidant glutathione by processes such as PDT, which leaves the highly cytotoxic radicals of Etop intact [66]. Thus, the effects of PpIX PDT and Etop-radicals are potentiated by the respective mechanisms mentioned previously, leading to synergy when both treatments are given together. The increase in both ROS and percentage of apoptotic cells treated with Etop/PpIX-PAMAM(G5) Ns phototherapy is consistent with the mechanisms described. A favorable dose reduction for Etop over PpIX by phototherapy was noted on the dose reduction tracings with or without PAMAM(G5) Ns as the delivery platform. This is because of the sub-lethal PDT dose of PpIX selected, which is only necessary to elicit the light-induced membrane-disruptive and membrane- permeabilization effects of PCI. Nanoparticles in general predominantly localize to the endo-lysosomal compartment and are destined for degradation, thus the therapeutic efficacy could even be negatively impacted in the absence of specific escape mechanisms [67]. Endosomal escape by the proton sponge effect has been described for dendrimers owing to the abundance of tertiary amines in its structure [68]. Intriguingly, the inclusion of Etop and PpIX within PAMAM(G5) Ns appeared to have an antagonistic effect in the dark at high doses. Although the mechanism requires further study, this finding is advantageous in terms of lowered drug dark toxicity and improved photo-selectivity by phototherapy.

Despite great advances in delivery systems based on nanocarriers, the lack of targeting capability and hence treatment specificity limits its application to the clinic. PCI represents an approach that enables greater spatial selectivity through trigger-activated cargo release, which is confined to the illuminated region. This could be achieved either by co-administering Ps with the drug-loaded nanoparticle or by incorporating Ps into the nanoparticle [69,70,71,72]. The latter approach not only localizes the Ps to the endosome but also simplifies the treatment regime. Various types of nanocarriers have been studied for PCI; for example, the work by Bettache et al. utilized periodic mesoporous ionosilica nanoparticles incoporating small interfering RNA (siRNA) and a porphyrin derivative to enable imaging, PDT, and PCI of the siRNA. The study found a remarkable PDT and gene-silencing effect following light irradiation on a breast cancer cell line [73]. In another report, photo- and pH-degradable nanoparticles carrying hematoporphyrin and camptothecin were made from polymers of the polyacetal family; an increased drug potency and specificity were found against HeLa cells and the treatment effect was attributed to the release of the cargo through photo-chemo-degradation of the nanoparticle within the endosome, followed by hematoporphyrin-mediated PDT and PCI of camptothecin [74]. The multi-functional potential of dendrimers as a nanocarrier fulfills the requirement for treatment using the PCI approach as it contains numerous surface functional groups and internal cavities for loading of pharmaceutics; for example, the study by Shieh et al. treated the human gingival cancer cell line Ca9-22 with doxorubicin conjugated to generation 4.5 PAMAM dendrimers and found the cytotoxicity was significantly improved from that of the free drug when the Ps disulfonated aluminum phthalocyanine was used for PCI in the “light after” drug regime [75]. In another study, Lai et al. investigated the influence of PCI on doxorubicin using polymeric micelles constructed with dendrimer-phthalocyanine and poly(ethylene)-*b*-poly(l-lysine) block copolymer on drug-resistant MCF-7 cells and a xenograft. The study demonstrated greater effectiveness deeper into the tumor than by PDT alone, which could better facilitate treatment of drug-resistant and deep-seated lesions previously thought unsuitable by PDT [76]. We envision that nanotechnology-based PCI would be best suited as an adjuvant to surgical resection of GBM, where disease control at the surgical margin could be better achieved by surface illumination of the resection cavity or even by interstitial illumination deep to the resection margin. The enhanced therapeutic effect of Etop on U87-MG human glioma cells triggered only by light using dendrimer Ns loaded with Etop and PpIX in the current study calls for further studies of efficacy and toxicity on animal models.

## 5. Conclusions

In this study, synergism between Etop and PpIX phototherapy in terms of the in vitro tumoricidal effect was demonstrated in favor of the Etop/PpIX-PAMAM(G5) Ns. This could be attributed to increased cellular uptake and endo-lysosomal targeting with subsequent PCI by the Etop/PpIX-PAMAM(G5) Ns. Mechanistically, both ROS and apoptosis are increased through this approach and additional drug-to-drug interactions specific to Etop other than PCI may be responsible, which warrants further study. The lowered dark toxicity associated with the Etop/PpIX-PAMAM(G5) Ns has the potential to reduce unwanted drug side effects and improve photo-selectivity for targeted phototherapy.

## Figures and Tables

**Table 1:** Average diameter, zeta potential, polydispersity index, and loading efficiency of the nanocarriers.

Nanocarrier	Dav	Zeta Potential	PDI	Loading Efficiency	
				PpIX	Etop
	(nm)	(mV)		(%)	
PAMAM(G5) Ns	74.8 ± 1.1	40.1 ± 0.4	0.09 ± 0.01	-	
Etop/PpIX-PAMAM(G5) Ns	217.4 ± 2.9	40.5 ± 1.3	0.24 ± 0.02	100	12

*N* = 6; *p* < 0.001. (Dav—average diameter, Etop—etoposide, Ns—nanosphere, PAMAM(G5)—generation-5 polyamidoamine dendrimer, PDI—polydispersity index, PpIX—protoporphyrin IX).

## Data Availability

Not applicable.
